# Supplementary material for: Acquiring Iron-Reducing Enrichment Cultures: Environments, Methods and Quality Assessments
Source: Microorganisms. 2023 Feb 10;11(2):448. doi: 10.3390/microorganisms11020448 (PMC9959475; doi:10.3390/microorganisms11020448)
Supplement: Supplementary file 1 [file microorganisms-11-00448-s001.zip › microorganisms-2065393-supplementary.pdf]

**Supplementary Material**

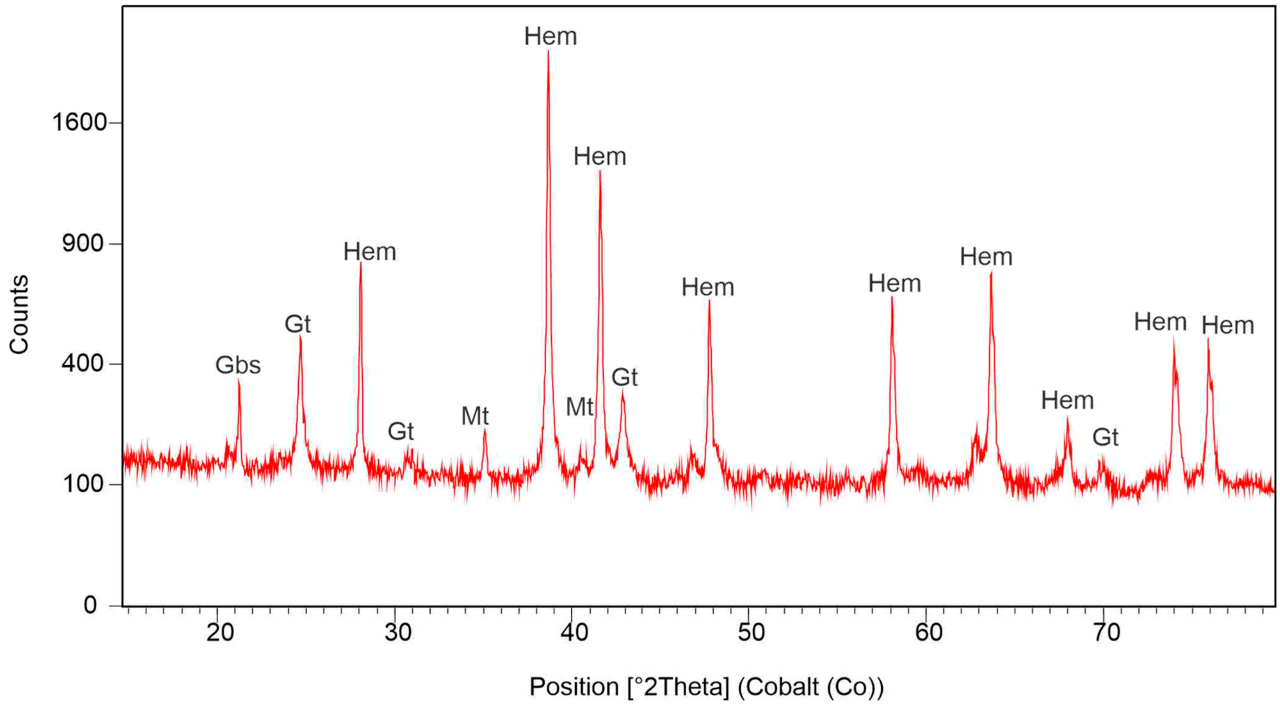


**Figure S1.** X-ray diffractogram of the canga sample used as an iron source in a bioreactor. Abrev. Hem: hematite; Gt: goethite; Mt: magnetite; Gbs: gibbsite.

**Table S1**. Geographic coordinates and habitat description of the collected inocula.

| Inoculum | Latitude | Longitude | Description |
|---|---|---|---|
| 1-SS | -6.3548 | -50.44867 | Small water pond on an outcropping lateritic duricrust |
| 2-SS | -6.3548 | -50.44869 | Organic mat at the margins of small creek on a *canga* plateau |
| 3-SS | -6.3558 | -50.44914 | Organic mat at the margins of a permanent *canga* lake |
| 4-SS | -6.3488 | -50.4427 | Periodically inundated grassland |
| 5-SS | -6.3735 | -50.38426 | Sediment rich in organic matter at the margin of a permanent *canga* lake |
| 6-SN | -6.0537 | -50.17793 | Plateau on the top of a iron mining waste pile |
| 7-SN | -6.0129 | -50.29743 | Sediment collected at the margins of a temporary *canga* lake |

**Table S2.** Cell growth of selected microbial inocula. Number of generations (n), generation time (g) and growth rate (k) of the selected inocula.

| Consortium | Time (h) | Total live/µL | n | g (h) | k |
|---|---|---|---|---|---|
| 4-SS | 0 | 1731,916529 | – | – | – |
| | 24 | 2592,100662 | 0,5779122 | 77,866501 | 0,0148437 |
| | 48 | 2953,07221 | 0,7647655 | 62,764341 | 0,0243694 |
| | 72 | 1952,812239 | 0,1720407 | 302,25414 | 0,0011384 |
| 6-SN | 0 | - | – | – | – |
| | 24 | 2636,845079 | 2,064803 | 10,170462 | 0,4060392 |
| | 48 | 4184,387394 | 2,8115646 | 17,783692 | 0,3161958 |
| | 72 | 3282,936337 | 2,3788898 | 23,540392 | 0,2021113 |

**Table S3**. Taxonomic annotations and relative abundance (%) of 16S rDNA OTUs detected in the selected iron-reducing microbial inocula 4-SS and 6-SN originating from *cangas* in the Carajás National Forest, Eastern Amazon, Brazil.

| Number | OTUId | 4-SS (%) | 6-SN (%) | Phylum | Class | Order | Family | Genus |
|---|---|---|---|---|---|---|---|---|
| 1 | OTU330588054 | 37.35 | 0.58 | Proteobacteria | Alphaproteobacteria | Rhizobiales | Rhizobiaceae | - |
| 2 | OTU778287863 | 21.37 | 3.23 | Proteobacteria | Gammaproteobacteria | Betaproteobacteriales | Burkholderiaceae | Achromobacter |
| 3 | OTU514564031 | 0.02 | 20.55 | Proteobacteria | Gammaproteobacteria | Enterobacteriales | Enterobacteriaceae | Serratia |
| 4 | OTU638450427 | 17.64 | 0.25 | Proteobacteria | Alphaproteobacteria | Rhizobiales | Rhizobiaceae | - |
| 5 | OTU327073807 | 0.00 | 9.44 | Proteobacteria | Gammaproteobacteria | Enterobacteriales | Enterobacteriaceae | Enterobacter |
| 6 | OTU664802110 | 7.76 | 1.19 | Proteobacteria | Gammaproteobacteria | Betaproteobacteriales | Burkholderiaceae | Achromobacter |
| 7 | OTU16479386 | 0.00 | 6.28 | Proteobacteria | Gammaproteobacteria | Enterobacteriales | Enterobacteriaceae | Serratia |
| 8 | OTU205309840 | 0.00 | 5.72 | Proteobacteria | Gammaproteobacteria | Enterobacteriales | Enterobacteriaceae | Serratia |
| 9 | OTU197713031 | 0.00 | 5.55 | Proteobacteria | Gammaproteobacteria | Enterobacteriales | Enterobacteriaceae | Serratia |
| 10 | OTU810266754 | 0.00 | 5.53 | Proteobacteria | Gammaproteobacteria | Enterobacteriales | Enterobacteriaceae | Serratia |
| 11 | OTU453394013 | 0.00 | 5.30 | Proteobacteria | Gammaproteobacteria | Enterobacteriales | Enterobacteriaceae | Serratia |
| 12 | OTU745384667 | 4.62 | 0.65 | Proteobacteria | Gammaproteobacteria | Betaproteobacteriales | Burkholderiaceae | Achromobacter |
| 13 | OTU983293026 | 0.00 | 4.82 | Proteobacteria | Gammaproteobacteria | Enterobacteriales | Enterobacteriaceae | Serratia |
| 14 | OTU361044378 | 4.31 | 0.00 | Proteobacteria | Gammaproteobacteria | Xanthomonadales | Xanthomonadaceae | Stenotrophomonas |
| 15 | OTU409692057 | 2.83 | 0.00 | Proteobacteria | Gammaproteobacteria | Xanthomonadales | Xanthomonadaceae | Stenotrophomonas |
| 16 | OTU29726137 | 0.00 | 2.46 | Proteobacteria | Gammaproteobacteria | Enterobacteriales | Enterobacteriaceae | - |
| 17 | OTU296052419 | 0.00 | 2.06 | Proteobacteria | Gammaproteobacteria | Enterobacteriales | Enterobacteriaceae | Enterobacter |
| 18 | OTU931675536 | 0.00 | 1.96 | Proteobacteria | Gammaproteobacteria | Enterobacteriales | Enterobacteriaceae | - |
| 19 | OTU315278921 | 0.00 | 1.87 | Proteobacteria | Gammaproteobacteria | Enterobacteriales | Enterobacteriaceae | - |
| 20 | OTU658386521 | 0.00 | 1.82 | Proteobacteria | Gammaproteobacteria | Enterobacteriales | Enterobacteriaceae | Escherichia |
| 21 | OTU47550649 | 0.00 | 1.78 | Proteobacteria | Gammaproteobacteria | Betaproteobacteriales | Burkholderiaceae | Comamonas |

| | | | | | | | | |
|---|---|---|---|---|---|---|---|---|
| 22 | OTU392786384 | 0.00 | 1.66 | Proteobacteria | Gammaproteobacteria | Enterobacteriales | Enterobacteriaceae | Serratia |
| 23 | OTU326136951 | 0.00 | 1.55 | Proteobacteria | Gammaproteobacteria | Enterobacteriales | Enterobacteriaceae | Serratia |
| 24 | OTU340260015 | 0.00 | 1.48 | Proteobacteria | Gammaproteobacteria | Enterobacteriales | Enterobacteriaceae | Serratia |
| 25 | OTU270599485 | 0.00 | 1.40 | Proteobacteria | Gammaproteobacteria | Enterobacteriales | Enterobacteriaceae | Serratia |
| 26 | OTU188582409 | 0.00 | 1.33 | Proteobacteria | Gammaproteobacteria | Enterobacteriales | Enterobacteriaceae | Serratia |
| 27 | OTU977738112 | 0.00 | 1.27 | Proteobacteria | Gammaproteobacteria | Enterobacteriales | Enterobacteriaceae | Serratia |
| 28 | OTU531857961 | 0.00 | 1.26 | Proteobacteria | Gammaproteobacteria | Enterobacteriales | Enterobacteriaceae | Serratia |
| 29 | OTU551794623 | 0.00 | 1.21 | Proteobacteria | Gammaproteobacteria | Betaproteobacteriales | Burkholderiaceae | Delftia |
| 30 | OTU679120852 | 0.00 | 1.19 | Proteobacteria | Gammaproteobacteria | Enterobacteriales | Enterobacteriaceae | Serratia |
| 31 | OTU188026163 | 0.07 | 1.01 | Proteobacteria | Gammaproteobacteria | Betaproteobacteriales | Burkholderiaceae | Achromobacter |
| 32 | OTU738426776 | 0.00 | 1.06 | Proteobacteria | Gammaproteobacteria | Enterobacteriales | Enterobacteriaceae | Serratia |
| 33 | OTU982667559 | 0.00 | 1.05 | Proteobacteria | Gammaproteobacteria | Enterobacteriales | Enterobacteriaceae | Serratia |
| 34 | OTU380615083 | 0.87 | 0.00 | Proteobacteria | Gammaproteobacteria | Xanthomonadales | Xanthomonadaceae | Stenotrophomonas |
| 35 | OTU292188812 | 0.74 | 0.03 | Proteobacteria | Gammaproteobacteria | Betaproteobacteriales | Burkholderiaceae | Achromobacter |
| 36 | OTU964900292 | 0.00 | 0.53 | Proteobacteria | Gammaproteobacteria | Enterobacteriales | Enterobacteriaceae | Enterobacter |
| 37 | OTU18097637 | 0.48 | 0.00 | Firmicutes | Bacilli | Lactobacillales | Enterococcaceae | Enterococcus |
| 38 | OTU366068798 | 0.00 | 0.41 | Proteobacteria | Gammaproteobacteria | Betaproteobacteriales | Burkholderiaceae | Delftia |
| 39 | OTU943463416 | 0.41 | 0.00 | Proteobacteria | Alphaproteobacteria | Rhizobiales | Rhizobiaceae | - |
| 40 | OTU520549349 | 0.00 | 0.39 | Proteobacteria | Gammaproteobacteria | Betaproteobacteriales | Burkholderiaceae | Delftia |
| 41 | OTU795369909 | 0.21 | 0.03 | Proteobacteria | Gammaproteobacteria | Betaproteobacteriales | Burkholderiaceae | Achromobacter |
| 42 | OTU135898228 | 0.23 | 0.00 | Proteobacteria | Alphaproteobacteria | Rhizobiales | Rhizobiaceae | Nitratireductor |
| 43 | OTU392193377 | 0.21 | 0.00 | Proteobacteria | Alphaproteobacteria | Rhizobiales | Rhizobiaceae | - |
| 44 | OTU414747809 | 0.19 | 0.00 | Proteobacteria | Alphaproteobacteria | Rhizobiales | Rhizobiaceae | Ensifer |
| 45 | OTU960888903 | 0.00 | 0.18 | Proteobacteria | Gammaproteobacteria | Enterobacteriales | Enterobacteriaceae | - |
| 46 | OTU376647409 | 0.00 | 0.15 | Proteobacteria | Gammaproteobacteria | Enterobacteriales | Enterobacteriaceae | Serratia |
| 47 | OTU147043709 | 0.14 | 0.00 | Proteobacteria | Alphaproteobacteria | Rhizobiales | Rhizobiaceae | - |

| | | | | | | | | |
|---|---|---|---|---|---|---|---|---|
| 48 | OTU271811333 | 0.00 | 0.12 | Proteobacteria | Gammaproteobacteria | Betaproteobacteriales | Burkholderiaceae | Cupriavidus |
| 49 | OTU911841220 | 0.10 | 0.02 | Proteobacteria | Gammaproteobacteria | Betaproteobacteriales | Burkholderiaceae | Achromobacter |
| 50 | OTU988281038 | 0.00 | 0.11 | Proteobacteria | Gammaproteobacteria | Enterobacteriales | Enterobacteriaceae | - |
| 51 | OTU732346435 | 0.00 | 0.09 | Proteobacteria | Gammaproteobacteria | Enterobacteriales | Enterobacteriaceae | Serratia |
| 52 | OTU547918392 | 0.00 | 0.09 | Proteobacteria | Gammaproteobacteria | Enterobacteriales | Enterobacteriaceae | Escherichia |
| 53 | OTU973640528 | 0.08 | 0.00 | Proteobacteria | Alphaproteobacteria | Rhizobiales | Rhizobiaceae | - |
| 54 | OTU781542215 | 0.00 | 0.08 | Proteobacteria | Gammaproteobacteria | Enterobacteriales | Enterobacteriaceae | Serratia |
| 55 | OTU824939365 | 0.00 | 0.07 | Proteobacteria | Gammaproteobacteria | Enterobacteriales | Enterobacteriaceae | Serratia |
| 56 | OTU814223033 | 0.00 | 0.07 | Proteobacteria | Gammaproteobacteria | Enterobacteriales | Enterobacteriaceae | Serratia |
| 57 | OTU334938039 | 0.07 | 0.00 | Proteobacteria | Gammaproteobacteria | Xanthomonadales | Xanthomonadaceae | Stenotrophomonas |
| 58 | OTU619830231 | 0.00 | 0.07 | Proteobacteria | Gammaproteobacteria | Enterobacteriales | Enterobacteriaceae | Serratia |
| 59 | OTU924800355 | 0.06 | 0.01 | Proteobacteria | Gammaproteobacteria | Betaproteobacteriales | Burkholderiaceae | Achromobacter |
| 60 | OTU758147632 | 0.06 | 0.01 | Proteobacteria | Gammaproteobacteria | Betaproteobacteriales | Burkholderiaceae | Achromobacter |
| 61 | OTU386313741 | 0.00 | 0.06 | Proteobacteria | Gammaproteobacteria | Enterobacteriales | Enterobacteriaceae | Serratia |
| 62 | OTU839269590 | 0.06 | 0.00 | Proteobacteria | Alphaproteobacteria | Rhizobiales | Rhizobiaceae | Ochrobactrum |
| 63 | OTU297544569 | 0.00 | 0.06 | Proteobacteria | Gammaproteobacteria | Enterobacteriales | Enterobacteriaceae | Serratia |
| 64 | OTU311483204 | 0.05 | 0.01 | Proteobacteria | Gammaproteobacteria | Betaproteobacteriales | Burkholderiaceae | Achromobacter |
| 65 | OTU652410420 | 0.00 | 0.06 | Proteobacteria | Gammaproteobacteria | Betaproteobacteriales | Burkholderiaceae | Acidovorax |
| 66 | OTU764472303 | 0.00 | 0.06 | Proteobacteria | Gammaproteobacteria | Enterobacteriales | Enterobacteriaceae | Serratia |
| 67 | OTU616058265 | 0.00 | 0.05 | Proteobacteria | Gammaproteobacteria | Enterobacteriales | Enterobacteriaceae | Serratia |
| 68 | OTU271450297 | 0.00 | 0.05 | Proteobacteria | Gammaproteobacteria | Enterobacteriales | Enterobacteriaceae | Serratia |
| 69 | OTU184462265 | 0.00 | 0.05 | Proteobacteria | Gammaproteobacteria | Enterobacteriales | Enterobacteriaceae | Serratia |
| 70 | OTU25917547 | 0.00 | 0.05 | Proteobacteria | Gammaproteobacteria | Enterobacteriales | Enterobacteriaceae | Serratia |
| 71 | OTU868684207 | 0.00 | 0.05 | Proteobacteria | Gammaproteobacteria | Enterobacteriales | Enterobacteriaceae | Serratia |
| 72 | OTU640213820 | 0.04 | 0.00 | Proteobacteria | Alphaproteobacteria | Rhizobiales | Rhizobiaceae | - |
| 73 | OTU535608073 | 0.04 | 0.00 | Proteobacteria | Alphaproteobacteria | Rhizobiales | Rhizobiaceae | - |

| | | | | | | | | |
|---|---|---|---|---|---|---|---|---|
| 74 | OTU206876812 | 0.00 | 0.04 | Proteobacteria | Gammaproteobacteria | Enterobacteriales | Enterobacteriaceae | Serratia |
| 75 | OTU840799738 | 0.00 | 0.04 | Firmicutes | Bacilli | Bacillales | Paenibacillaceae | Paenibacillus |
| 76 | OTU563053320 | 0.00 | 0.03 | Proteobacteria | Gammaproteobacteria | Enterobacteriales | Enterobacteriaceae | Serratia |
| 77 | OTU729182799 | 0.00 | 0.03 | Proteobacteria | Gammaproteobacteria | Enterobacteriales | Enterobacteriaceae | Serratia |
| 78 | OTU326369350 | 0.00 | 0.03 | Proteobacteria | Gammaproteobacteria | Enterobacteriales | Enterobacteriaceae | Enterobacter |
| 79 | OTU970582418 | 0.00 | 0.03 | Proteobacteria | Gammaproteobacteria | Enterobacteriales | Enterobacteriaceae | - |
| 80 | OTU945880892 | 0.00 | 0.03 | Proteobacteria | Gammaproteobacteria | Enterobacteriales | Enterobacteriaceae | Serratia |
| 81 | OTU441838924 | 0.00 | 0.03 | Proteobacteria | Gammaproteobacteria | Enterobacteriales | Enterobacteriaceae | Serratia |
| 82 | OTU612862832 | 0.00 | 0.03 | Proteobacteria | Gammaproteobacteria | Enterobacteriales | Enterobacteriaceae | Enterobacter |
| 83 | OTU506960675 | 0.00 | 0.03 | Proteobacteria | Gammaproteobacteria | Enterobacteriales | Enterobacteriaceae | Serratia |
| 84 | OTU866585139 | 0.00 | 0.03 | Proteobacteria | Gammaproteobacteria | Enterobacteriales | Enterobacteriaceae | Trabulsiella |
| 85 | OTU251620561 | 0.00 | 0.03 | Proteobacteria | Gammaproteobacteria | Enterobacteriales | Enterobacteriaceae | Enterobacter |
| 86 | OTU820552923 | 0.00 | 0.03 | Proteobacteria | Gammaproteobacteria | Enterobacteriales | Enterobacteriaceae | Serratia |
| 87 | OTU418915799 | 0.00 | 0.02 | Proteobacteria | Gammaproteobacteria | Enterobacteriales | Enterobacteriaceae | Enterobacter |
| 88 | OTU989969933 | 0.00 | 0.02 | Proteobacteria | Gammaproteobacteria | Enterobacteriales | Enterobacteriaceae | Serratia |
| 89 | OTU995121149 | 0.00 | 0.02 | Proteobacteria | Gammaproteobacteria | Enterobacteriales | Enterobacteriaceae | Serratia |
| 90 | OTU974408404 | 0.00 | 0.02 | Proteobacteria | Gammaproteobacteria | Enterobacteriales | Enterobacteriaceae | Serratia |
| 91 | OTU861900632 | 0.00 | 0.02 | Proteobacteria | Gammaproteobacteria | Enterobacteriales | Enterobacteriaceae | Serratia |
| 92 | OTU911930897 | 0.00 | 0.02 | Proteobacteria | Gammaproteobacteria | Betaproteobacteriales | Burkholderiaceae | Burkholderia |
| 93 | OTU628461955 | 0.00 | 0.02 | Proteobacteria | Gammaproteobacteria | Enterobacteriales | Enterobacteriaceae | Serratia |
